# Benchmarking of force fields to characterize the intrinsically disordered R2-FUS-LC region

**DOI:** 10.1038/s41598-023-40801-6

**Published:** 2023-08-30

**Authors:** Maud Chan-Yao-Chong, Justin Chan, Hidetoshi Kono

**Affiliations:** 1 Molecular Modeling and Simulation (MMS) Team, Institute for Quantum Life Science, National Institutes for Quantum Science and Technology (QST), 4-9-1, Anagawa, Inage Ward, Chiba City, Chiba 263-8555 Japan; 2 Present Address: Toulouse Biotechnology Institute, TBI, Université de Toulouse, CNRS, INRAE, INSA, 135, Avenue de Rangueil, 31077 Toulouse Cedex 04, France

**Keywords:** Biochemistry, Protein folding, Biophysics, Molecular biophysics

## Abstract

Intrinsically Disordered Proteins (IDPs) play crucial roles in numerous diseases like Alzheimer's and ALS by forming irreversible amyloid fibrils. The effectiveness of force fields (FFs) developed for globular proteins and their modified versions for IDPs varies depending on the specific protein. This study assesses 13 FFs, including AMBER and CHARMM, by simulating the R2 region of the FUS-LC domain (R2-FUS-LC region), an IDP implicated in ALS. Due to the flexibility of the region, we show that utilizing multiple measures, which evaluate the local and global conformations, and combining them together into a final score are important for a comprehensive evaluation of force fields. The results suggest c36m2021s3p with mTIP3p water model is the most balanced FF, capable of generating various conformations compatible with known ones. In addition, the mTIP3P water model is computationally more efficient than those of top-ranked AMBER FFs with four-site water models. The evaluation also reveals that AMBER FFs tend to generate more compact conformations compared to CHARMM FFs but also more non-native contacts. The top-ranking AMBER and CHARMM FFs can reproduce intra-peptide contacts but underperform for inter-peptide contacts, indicating there is room for improvement.

## Introduction

Intrinsically disordered proteins (IDPs) are proteins that can form different conformations depending on the environment and their binding partners^1^. Some IDPs can self-aggregate to form amyloid fibrils which take on the cross-β structure^2^. The cross-β structure consists of beta-strand proteins/peptides that are stacked along the length of the fiber forming long beta-sheets called protofibril. Finally, complexes of protofibrils form amyloid fibrils^3^.

Amyloid fibrils are associated with diseases^4,5^ such as Alzheimer’s, Parkinson’s, type II diabetes, Amyotrophic Lateral Sclerosis (ALS)^4,6,7^ and others. ALS is a rare neurodegenerative disease^8,9^ where in 50% of cases, death occurs within three years of the first clinical manifestation^10^. In ALS patients, amino acid mutations have been found in the Low-Complexity (LC) region of the Fused in Sarcoma (FUS) protein^11–16^. Irreversible amyloid fibril aggregation has been observed in mutated FUS-LC region, whereas reversible fibrils are observed in the wild-type^11,16,17^.

**Figure 1:**
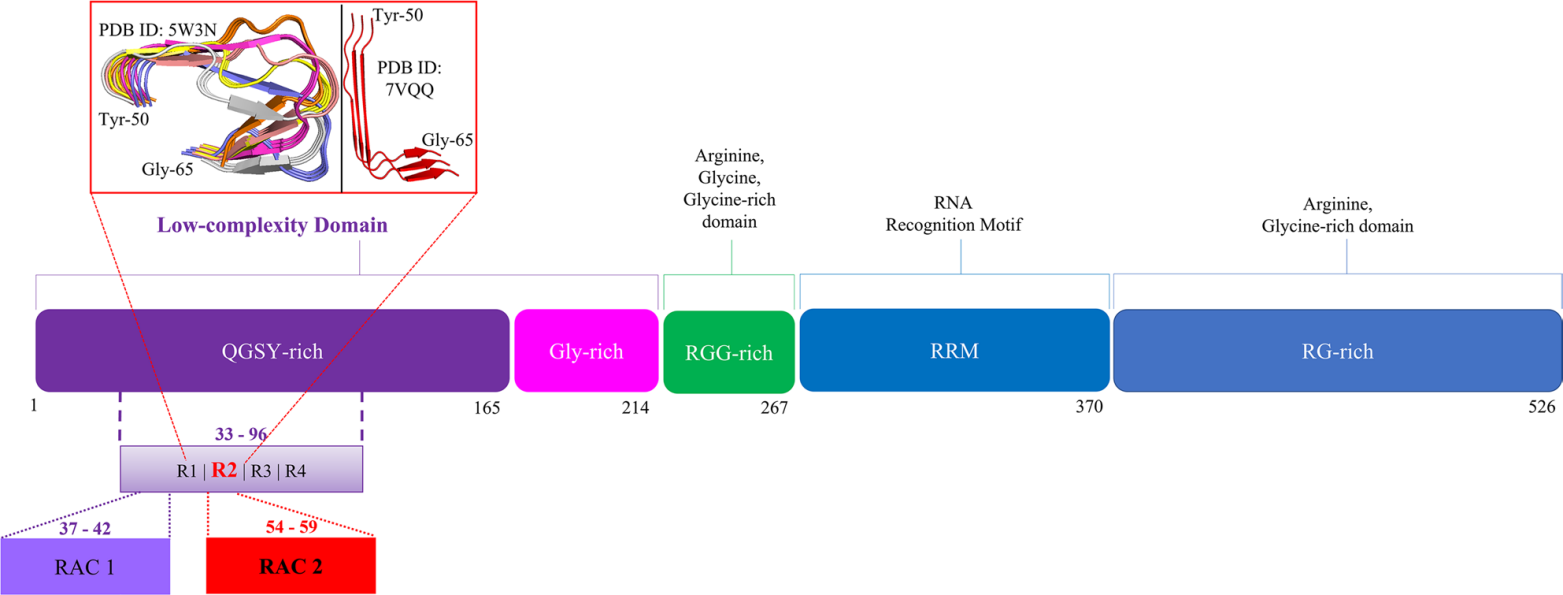
Domain organization of the full length Fused in Sarcoma (FUS) protein. The FUS N-terminal low-complexity (LC) domain (residues 1–214) contains a QGSY-rich prion-like domain (1–165, violet box) and a Gly-rich region (166–214, pink box). Within the QGSY-rich domain, there are four repeat motifs (R1–R2–R3–R4). Within R2 (R2-FUS-LC region) there is a Reversible Amyloid Fibril Core (RAC 2) that is involved in fibril formation. We take only the R2-FUS-LC region to study FUS fibrillation. Inside on the top red square are two different experimentally solved conformations of the R2-FUS-LC region. On the left, six representatives of R2-FUS-LC region selected from the 20 models of the NMR structure (PDB ID: 5W3N^16^, “U-shaped”). On the right, the cryo-EM model from PDB ID: 7VQQ^24^, “L-shaped”. Figure was prepared with Microsoft PowerPoint and VMD v1.9.3^25^ (https://www.ks.uiuc.edu/Research/vmd/).

The human FUS protein (526 residues) is involved in mRNA splicing and transcription. The FUS-LC-core_33–96_ is involved in amyloid fibril formation^11,17–21^ and contains four repeat motifs (R1-R2-R3-R4)^17^ (Fig. S1, violet boxes). Within R1/R2, the tandem [S/G]Y[S/G] motifs have been implicated in the formation of Reversible Amyloid fibril Cores (RAC)^17^ (Fig. 1 and Fig. S1). The R2 region has been known to be more important for fibril formation than R1^22,23^. The structures of the FUS-LC-core_33–96_^16,24^ (Fig. 1) show the R2 region has few long-distance contacts with the rest of the LC-core domain (Fig. S2). Therefore, the R2-FUS-LC_50–65_ region is a good candidate for studying amyloid fibrillation.

Our current understanding of the mechanism of amyloid fibril aggregation is poor. Furthermore, some of the transient intermediate structures of IDPs have been reported to be toxic^26^. One approach to study fibril formation and its intermediates is by performing all-atom Molecular Dynamics (MD) simulations. Amyloid β-peptides that form the amyloid fibrils in Alzheimer’s are commonly studied using MD simulations^27–29^.

However, experimental data and simulation results often show discrepencies^27,30^. One of the main reasons for this is that the all-atom force fields (FFs) such as AMBER, CHARMM^31,32^, OPLS-AA^33,34^ and GROMOS^35,36^ were developed to reproduce the properties of stable globular proteins. In contrast, IDPs adopt multiple unstable conformations where nonpolar residues are often exposed to the solvent. Multiple research groups have tuned FFs to better reproduce experimental data^27,37–42^ [Table S1 and Sup. Info. Section Benchmark of Force Fields (FFs) and Water Models (WMs)]. It is unclear if these modified FFs are generalizable across all IDPs. Note that throughout the text, when we refer to the FFs’, we are referring to the result or properties observed from performing simulations with the respective FF.

In this work, we apply widely used MD force fields for studying IDPs to the R2-FUS-LC (Fig. 1 and Fig. S1) to evaluate whether they can sample conformational ensembles of fibrils that are consistent with experimental data. We select ten FFs and water models (WMs) recently developed for IDPs. For comparison, we included c27s3p, a99sb4pew and a14sb3p FFs which were used to develop some of the IDP FFs. We score these thirteen FFs based on three criteria: the compactness of the fibrils, the intra-peptide contacts in the cross-β state and the secondary structure propensity. Surprisingly, most FFs fail to reproduce the experimental data. Our scoring method suggests that CHARMM36m2021 FF with the mTIP3P water model is the best for studying R2-FUS-LC fibrillation.

## Results

We conducted six MD simulations, each lasting 500 ns, totaling 3 μs, with each of thirteen FFs (Table S1). To evaluate the FFs against the reversible amyloid fibril R2-FUS-LC region (trimer of 16 residue peptides), we employed three measures: radius of gyration (Rg), secondary structure propensity (SSP) and intra-peptide contact map. Rg measures the global compactness/extension of the trimer and individual peptides (Fig. S3). SSP and intra-peptide contact map both concern the local contact details of the R2-FUS-LC region. We ranked the thirteen FFs based on a combined score derived from these three measures (Table 1, Final Score).

**Table 1:** Force field evaluation: summary of scores and measures.

Force fields	Rg score	SSP score	Contact map score	Final Scoreª	RANKING
c36m2021s3p*	1	0.65613	0.77657	5.10E−01	1
a99sb4pew*	0.39845	1	0.64988	2.59E−01	2
c36ms3p*	0.8692	0.31996	0.85621	2.38E−01	3
a19sbopc*	0.30691	0.61874	0.7161	1.36E−01	4
a99disp^•^	0.21986	0.51499	0.543	6.15E−02	5
a99sbildn4pd^•^	0.19783	0.45716	0.47616	4.31E−02	6
a99sbCufix3p^•^	0.07391	0.7345	0.34292	1.86E−02	7
c22s3p^•^	0.05156	0.4001	0.53323	1.10E−02	8
a03ws^#^	0.02029	0.10312	0.1012	2.12E−04	9
a14sb3p^#^	0.00001	0.76161	0.40067	3.05E−06	10
c36m3pm^#^	0.00029	0.0032	0.9946	9.23E−07	11
c36m2021s3pm^#^	0.04438	0.00001	1	4.44E−07	12
c27s3p^#^	0.0313	0.00001	0.00001	3.13E−12	13

ªObtained by multiplication of the three normalized Rg, SSP and contact map scores. The raw Rg, SSP and contact map scores were normalized by linearly rescaling them between 0.0001 to 1 (see “Methods” for details). *Rg* Radius of gyration, *SSP* Secondary Structure Propensity.

*Top ranking group.

^•^Middle ranking group.

^#^Bottom ranking group.

Based on the final score (Table 1), the thirteen FFs can be separated into three distinct groups: top (“*”), middle (“**•**”) and bottom (“**#**”) ranking groups according to the order of scores.

FFs in the “top” group have medium (0.3–0.7) to high (> 0.7) scores for all measures. FFs in the “bottom” group like c27s3p and a03ws, have low scores (< 0.3) in all three measures. However, a14sb3p stands out with relatively good scores for SSP and contact map, but a low Rg score. On the other hand, c36m3pm has the best intra-peptide contact map score but poor SSP score. FFs in “middle” ranking group tend to have low scores for at least one of the three measures but have medium agreement for the remaining. Details of the three measures will be explained in the following sections.

### Global compactness of R2-FUS-LC tripeptides

The Rg score measures the ability of the FFs to sample both compact and unfolded states of the R2-FUS-LC region. The reference data for the folded cross-β structure state comprises two distinct conformations of the R2-FUS-LC region: “U-shaped” conformation (PDB: 5W3N^16^) and “L-shaped” conformation (PDB: 7VQQ^24^), as shown in Fig. 1. Both conformations form a cross-β structure. Twenty U-shaped models with different loop conformations were solved by NMR with an average Rg of 10.0 Å (trimer of R2-FUS-LC) and the less compact (Rg: 14.4 Å) L-shaped conformation (trimer of R2-FUS-LC) was solved by cryo-EM. The reversibility of the cross-β structure^11,17^ suggests that the R2-FUS-LC region could adopt the unfolded state. To estimate the Rg of the unfolded state, we employed Flory's random coil polymer model with optimized parameters for IDPs^43^. We thus have three different measures of Rg: L-shaped, U-shaped, and Unfolded Rg’s.

**Figure 2:**
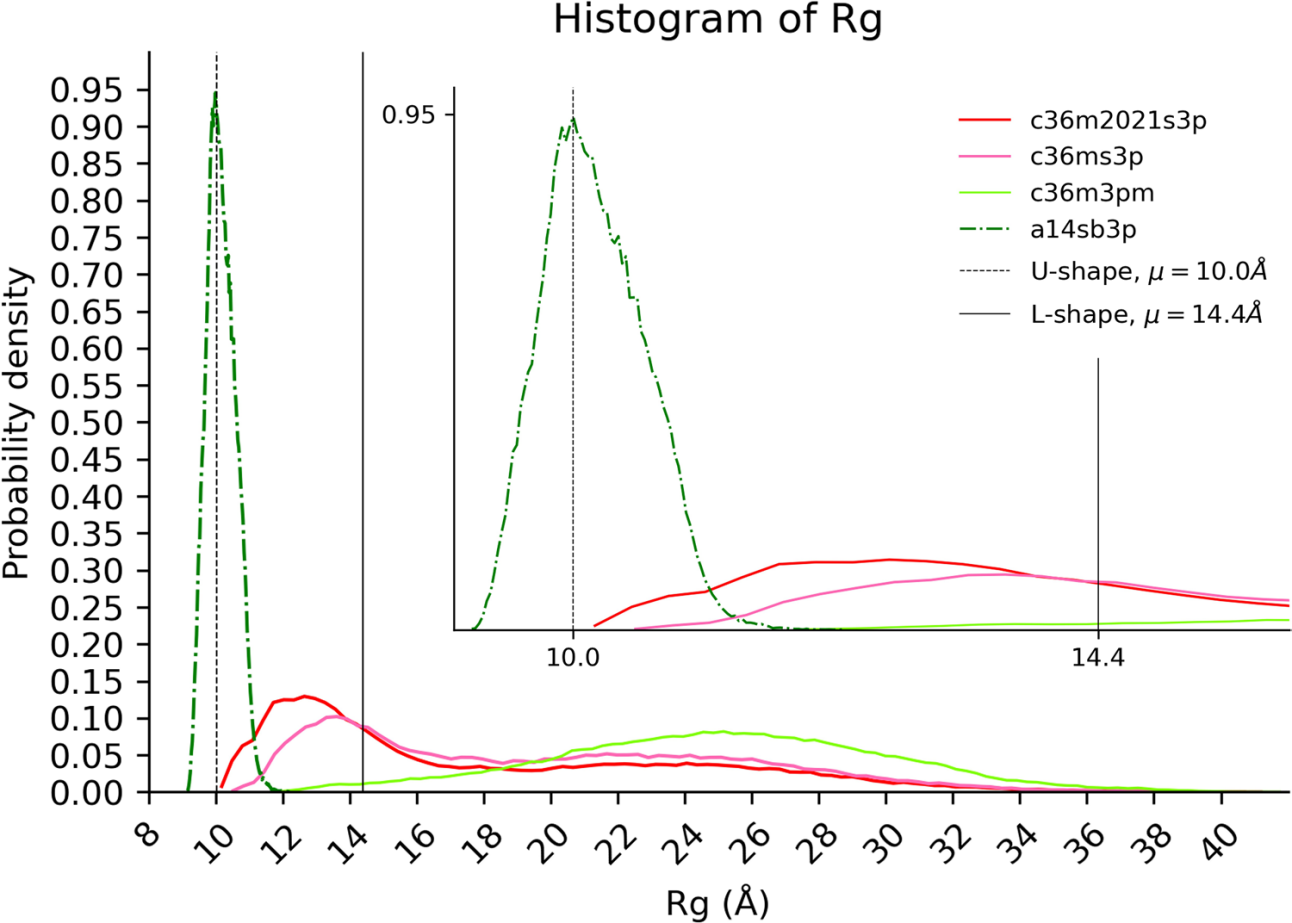
Distribution of the Radius of gyration (Rg). The top two ranking FFs for the final Rg score (Table 2) are C36m2021s3p (red) and c36ms3p (pink). These FFs can generate compact and extended conformations covering both the U and L-shaped Rg’s. However, a14sb3p (green) sampled compact conformations only (shown in the inset for a zoomed-in view). On the other hand, c36m3pm (light green) generated extended conformations. Both FFs rank at the bottom. Figure was prepared with Matplotlib v3.5^44^ (https://matplotlib.org/).

The distributions of Rg for snapshots from the simulation data are presented in Fig. 2 and Fig. S4. In order to assess how frequently the FFs can generate conformations that closely resemble the reference structures in terms of Rg, we fitted the Rg distributions with two Gaussian distributions. The distance from the *i*’th mean to the *k*’th reference Rg was computed as the absolute number of standard deviations (${\text{Z-score}}_{FF,k,i}$) of the *i*’th Gaussian. The lowest ${\text{Z-score}}_{FF,k,i}$ was chosen, inverted and normalized by linearly scaling it from 0.00001 to 1.0 (Table 2). The final Rg score was calculated by multiplying the three normalized scores and rescaling them in the same manner (Table 2).

**Table 2:** Normalized radius of gyration (Rg) scores for the 13 force fields.

Force fields	Normalized Rg score (rank)			Final Rg Scoreª
	U-shaped	L-shaped	Unfolded	
c36m2021s3p*	0.05654 (8)	0.26227 (3)	0.04817 (3)	1.00000
c36ms3p*	0.02194 (11)	1.00000 (1)	0.02830 (4)	0.86920
a99sb4pew*	1.00000 (1)	0.43082 (2)	0.00066 (12)	0.39845
a19sbopc*	0.11252 (4)	0.17544 (6)	0.01111 (5)	0.30691
a99disp^•^	0.09730 (5)	0.16614 (7)	0.00972 (7)	0.21986
a99sbildn4pd^•^	0.06440 (7)	0.20090 (5)	0.01092 (6)	0.19783
a99sbCufix3p^•^	0.14855 (3)	0.21916 (4)	0.00162 (11)	0.07391
c22s3p^•^	0.04650 (9)	0.11735 (9)	0.00675 (8)	0.05156
c36m2021s3pm^•^	0.00035 (12)	0.08977 (12)	1.00000 (1)	0.04438
c27s3p^#^	0.07513 (6)	0.13992 (8)	0.00213 (10)	0.03130
a03ws^#^	0.03703 (10)	0.10192 (10)	0.00384 (9)	0.02029
c36m3pm^#^	0.00001 (13)	0.08983 (11)	0.22404 (2)	0.00029
a14sb3p^#^	0.91433 (2)	0.00001 (13)	0.00001 (13)	0.00001

ªObtained by multiplication of the three normalized Rg scores and renormalizing them to 0.00001–1.0. FFs are sorted by the final Rg score in descending order.

*, ^•^ and ^#^: Same as in Table 1.

The thirteen FFs can be separated into three groups. Comparing the ranking of the final score (Table 1) and the final Rg score (Table 2), the top four FFs (c36m2021s3p, a99sb4pew, a19sbopc and c36ms3p) are the same although the order is slightly different.

Analyzing Table 2, we identified two distinct types of FFs among the top four. The first type includes c36m2021s3p and a19sbopc, both of which exhibited consistent performance across the three reference Rg’s. On the other hand, the second type consists of c36ms3p and a99sb4pew, which demonstrated a preference for flexible and compact conformations, respectively. We also observed bias towards the U-shaped Rg in a99sb4pew, a99sbCufix3p, and a14sb3p (Fig. S4), while c36m2021s3pm and c36m3pm (Fig. S4) favored the unfolded Rg. However, it is worth noting that the final Rg rank of these FFs is inversely correlated with the strength of their bias of sampled conformations. This is because our scoring scheme penalizes FFs that fit well to only one specific reference Rg but perform poorly for the others. As a result, the two worst-performing FFs are c36m3pm and a14sb3p (Fig. 2).

Overall, CHARMM FFs tend to generate more extended conformations than AMBER FFs (Figs. S3 and S4), except for a03ws.

Rg is a measure of the global compactness of a conformation, but it is not suitable for evaluating the details of the conformation. To assess the sampled conformations in more detail, we introduced two measures in the next sections: the intra-peptide contact map and the secondary structure propensity (SSP). These measures evaluate the intra-peptide interactions.

### Intra-peptide contacts of the R2-FUS-LC region

**Figure 3:**
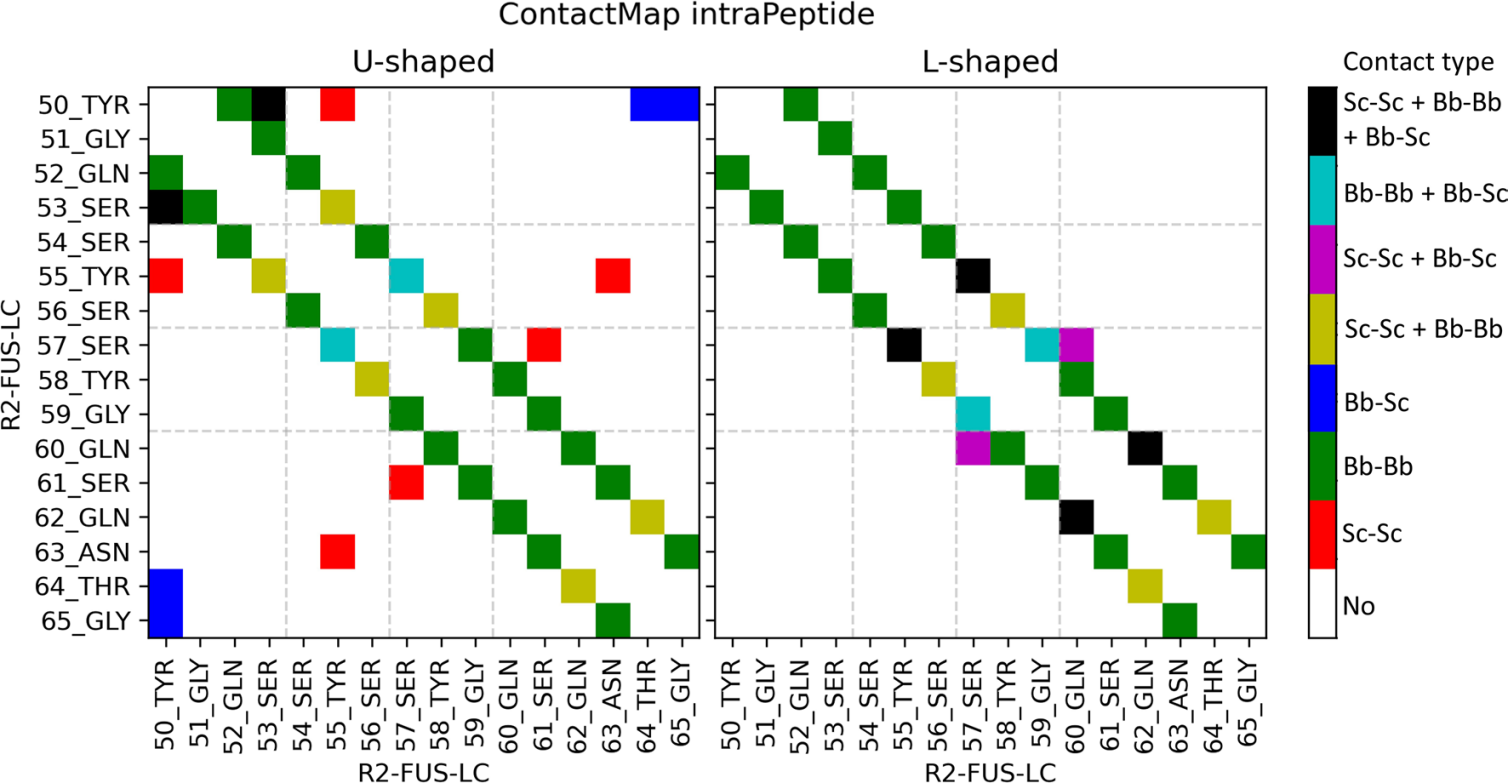
Intra-peptide contact map for U-shaped (left, PDB ID: 5W3N) and L-shaped (right, PDB ID: 7VQQ) conformations with a 5 Å cutoff. Contact types are color-labeled, with "Sc" corresponding to Side Chain and "Bb" to Backbone. In both conformations, the majority of contacts occur within ± 3 residues. The U-shaped conformation exhibits a few medium-distance contacts, whereas the L-shaped conformation lacks any medium-distance contacts. Figure was prepared with Matplotlib v3.5^44^ (https://matplotlib.org/).

The U-shaped and L-shaped conformations contain 20 and 15 intra-peptide contacts, respectively (Fig. 3). In the L-shaped conformation, there were no observed contacts between residues *j* and > *j* + 5 (medium-distance contacts) within a 5 Å cutoff. However, in the U-shaped conformation, medium-distance contacts are found between Tyr_50_–Tyr_55_, Tyr_50_–Thr_64_, Tyr_50_–Gly_65_, Tyr_55_–Asn_63_ and Ser_57_–Ser_61_. Therefore, we will only consider the U-shaped conformation for evaluating the FFs.

For each of the thirteen FF’s MD simulations, we calculated the average intra-peptide contacts for the trimers across all snapshots. The contacts from the FFs and the U-shaped conformation were compared using the Matthews Correlation coefficient (MCC)^45^ score (Table 3). The MCC score ranges from − 1 to 1, with a value of 1 indicating perfect correlation, − 1 indicating perfect anti-correlation, and 0 indicating no correlation. MCC scoring penalizes false predictions, which means that FFs that predicted the most native contacts (True Positives, TP), such as a99sb4pew, c27s3p, and a14sb3p (Table S2), may not necessarily be the top scoring FFs (Table 3). The process of normalization was carried out in a similar manner as described in the previous section.

**Table 3:** Matthews correlation coefficient (MCC) score of intra-peptide contact maps between force fields and the U-shaped conformation.

Force fields	Contact map score: MCC (U-shaped)	Norm. Contact Map Score
c36m2021s3pm^•^	0.70418	1.00000
c36m3pm^#^	0.70323	0.99460
c36ms3p*	0.67892	0.85621
c36m2021s3p*	0.66494	0.77657
a19sbopc*	0.65431	0.71610
a99sb4pew*	0.64268	0.64988
a99disp^•^	0.62391	0.54300
c22s3p^•^	0.62219	0.53323
a99sbildn4pd^•^	0.61217	0.47616
a14sb3p^#^	0.59891	0.40067
a99sbCufix3p^•^	0.58877	0.34292
a03ws^#^	0.54631	0.10120
c27s3p^#^	0.52854	0.00001

*, ^**•**^ and ^**#**^: Same as in Table 1.

In the contact map score ranking (Table 3), there are some surprising results. Despite being classified as a bottom performer based on the final score (Table 1), the c36m2021s3pm and c36m3pm FFs were ranked at the top in the contact map score. On the other hand, c36m2021s3p, which had the highest final score, was only ranked 4th. Additionally, a99sbCufix3p was placed at the bottom even though it had middle ranking in the final score.

Upon examining the confusion matrices for the four FFs (Table 4 and Table S2), we have a few observations. Firstly, c36m2021s3pm, c36m3pm, and c36m2021s3p, which have the highest Unfolded Rg scores, tend to favor more extended conformations. This is evident from the fact that < 20% of residues pairs are in contact. In contrast, a99sbCufix3p prefers more compact conformations, with 23.16% of residue pairs in contact.

**Table 4:** Confusion matrices of the intra-peptide contact maps for four FFs.

c36m2021s3p		Ref		Total Pred	a99sbCufix3p		Ref		Total Pred
		T	F				T	F	
Pred	T	13.86	**5.13**	18.99	Pred	T	14.17	**8.99**	23.16
	F	5.18	75.82	81.01		F	4.88	71.96	76.84

c36m2021s3pm		Ref		Total Pred	c36m3pm		Ref		Total Pred
		T	F				T	F	
Pred	T	13.75	**3.52**	17.27	Pred	T	13.75	**3.57**	17.32
	F	5.3	77.43	82.73		F	5.29	77.38	82.67

T: True/In contact (%).

F: False/No contact (%).

Bolded: False Positives (FP) or non-native contacts.

It is worth noting that while CHARMM FFs with extended conformations have fewer overall contacts (Table 4, Total Pred. True), they also have significantly lower non-native contacts (Table 4, bolded). Conversely, a99sbCufix3p has more contacts overall, but a larger proportion of these contacts are non-native (Table 4). After excluding these four FFs, the contact map score rankings of the remaining FFs (Table 3) were found to be consistent with the final score rankings (Table 1).

The L-shaped conformation primarily consists of β-strands but does not form β-sheets within the peptide. As a result, its conformations cannot be determined from the intra-peptide contact maps. This motivates us to assess the Secondary Structure Propensity (SSP) of these FFs in the following section.

### Secondary structure propensity (SSP) of R2-FUS-LC region

In each snapshot, Dictionary of Protein Secondary Structure (DSSP)^46,47^ is used to assign the secondary structure type (α-helix, β-strand, or coil) to each residue. For each residue, we define its SSP/probability for all secondary structure types by counting the number of occurrences in all snapshots and dividing it with the total number of snapshots. To evaluate the FFs’ SSP for the R2-FUS-LC region, we define the SSP score as the log likelihood of observing the experimental (U-shaped and L-shaped) secondary structures given the SSP probability distributions obtained from the simulation snapshots (details in Methods). Normalization was performed in a similar fashion as outlined previously.

In Table 5, we observe that the SSP score is higher for a14sb3p and a99sbCufix3p, but lower for c36ms3p and c36m2021s3pm compared to their final rank. The FFs with higher SSP scores tend to produce more compact conformations, as evidenced by their Rg scores (Table 2). Conversely, the FFs with lower rankings tend to favor more extended conformations. This suggests that the formation of native secondary structures is necessary for the development of compact fibrils.

**Table 5:** Normalized secondary structure propensity (SSP) scores.

Force fields	SSP score U-shaped	SSP score L-shaped	SSP Score
a99sb4pew*	1	1	1
a14sb3p^#^	0.852963	0.892893	0.761607
a99sbCufix3p^•^	0.823343	0.892089	0.734498
c36m2021s3p*	0.787453	0.833228	0.656131
a19sbopc*	0.725248	0.853141	0.618743
a99disp^•^	0.708013	0.72737	0.514992
a99sbildn4pd^•^	0.677607	0.674657	0.457158
c22s3p^•^	0.630039	0.635035	0.400103
c36ms3p*	0.564177	0.567119	0.319962
a03ws^#^	0.330989	0.311533	0.103123
c36m3pm^#^	0.044558	0.071552	0.003198
c36m2021s3pm^•^	0.00001	0.057822	0.00001
c27s3p^#^	0.011461	0.00001	0.00001

*, ^•^ and ^#^: Same as in Table 1.

**Figure 4:**
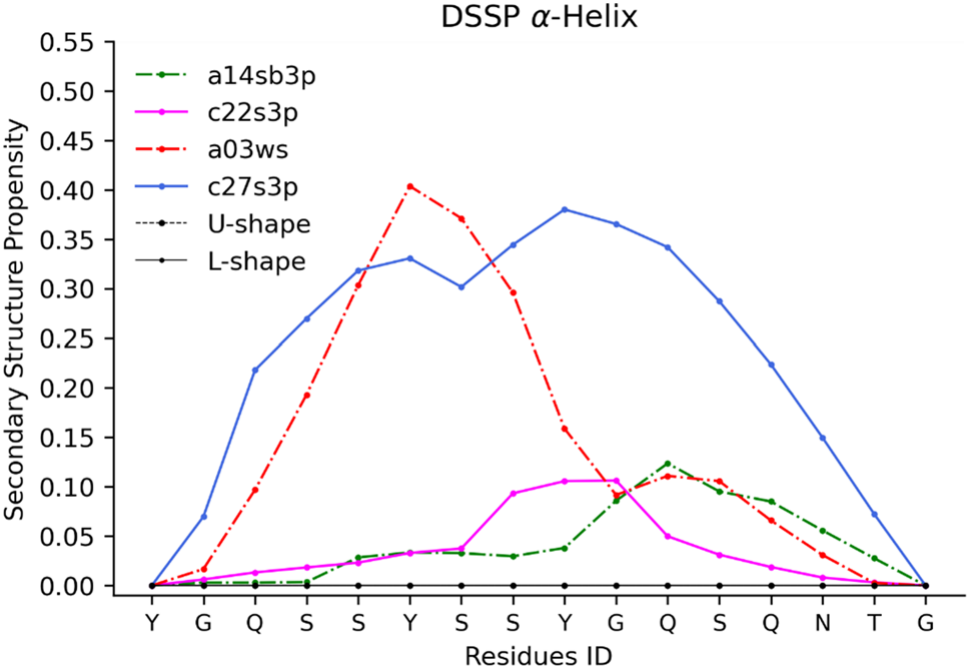
α-Helix Secondary Structure Propensity (SSP). Experimental data from Murray et al. indicate no α-helix propensity within the 16 residues. Only FFs with a per residue α-helix propensity greater than 0.1 are displayed. All FFs, except for a14sb3p (2nd ranked), are located at the bottom of the SSP rank (Table 5). Despite having a small amount of α-helix SSP, a14sb3p has a high SSP ranking due to its high β-strand SSP (Fig. S5). Figure was prepared with Matplotlib v3.5^44^ (https://matplotlib.org/).

The a03ws and c27s3p FFs, which have the lowest SSP scores, exhibit a strong tendency to generate conformations with α-helices around the RAC2 motif (Fig. 4). However, in both the L-shaped^24^ and U-shaped^16^ conformations, this region forms β-strands. NMR chemical shift data also confirms the absence of α-helices in the R2-FUS-LC region^16–18,22,48^.

## Discussion

Our observation indicates that AMBER FFs, specifically a14sb3p and a99sbCufix3p, generate more compact conformations compared to the CHARMM FFs, c36ms3p and c36m2021s3pm, which tend to produce more extended conformations. FFs that have more extended conformations can better replicate the properties of both U- and L-shaped conformations when compared to FFs generating more compact conformations that fit well only to one of these conformations.

Both AMBER and CHARMM FFs do not properly reproduce the inter-peptide interactions required to form fibrils (Table S3), although AMBER FFs perform relatively better than CHARMM FFs. The preference of CHARMM FFs for extended conformations extends to their inter-peptide interactions: the peptides spend approximately half the time as dimers or monomers in contrast to AMBER FFs (except a03ws) which mainly stay as trimers (Table S4). The inter-peptide interaction scores were not included in the final score as all FFs perform poorly and would just add noise to the final ranking.

In our study, the limited sampling time (only 3 µs) may have contributed to the poor performance observed. Fibril formation typically occurs over a much longer timescale of hours to days^16,24^. To alleviate but not resolve this issue, we have increased the peptide concentration from 0.16 mM (as used in NMR structure determination^16^) to 10 mM by decreasing the ratio of the number of peptides to the number of water molecules (add more waters) and initiated simulations from the U-shaped conformation in fibril form. Higher concentration of FUS peptides increases the chance of these peptides interacting with one another and forming fibrils. Additionally, we observed that most FF simulations have nearly converged within 300 ns by monitoring the average Rg values over time (Fig. S6).

It is possible that the chosen fragment is not capable of forming fibrils independently. To investigate this possibility, we analyzed the intra-protein interactions of the full-length LC domain (residues 1–214). The contact maps show that there are limited long-distance contacts between the R2-FUS-LC regions and the rest of the protein in the U/L-shaped conformations (Fig. S2), indicating that the R2-FUS-LC region functions as a distinct domain within the larger LC domain, at least in the fibril.

Our study corroborates previous research conducted by Lao et al. who utilized the a99sbildn4pd with TIP3P water model instead of TIP4PD to simulate a longer region of FUS that included the R2-FUS-LC region^23^. Our findings show similar contact map patterns as their study, and we also observed ~ 5% propensity of α-helices, which is consistent with their work. However, we noted a discrepancy in the β-strand propensity between our study and theirs, with their study observing much higher β-strand propensity than we did (Fig. S5). This difference may be attributed to the length of the peptide used in each study, with their study using a peptide length of 60 residues compared to our study's length of 16 residues.

Our findings are also consistent with other studies on Amyloid-β proteins, where different FFs produced different conformational ensembles, some of which are compatible with fibril aggregation^42,49,50^. For example, Pedersen et al. found that a19sbopc produces more compact conformations and forms more β-strand than a99disp^49^, while Samantray et al. demonstrated c36ms3p gave promising results for sampling and giving conformational ensembles with random coil and β-strand structures^50^. Finally, our final score ranking is the same as the Amyloid-β peptide (Aβ40) results from Robustelli et al. showing that c36ms3p is the best, except for c22s3p which underperformed a99disp and a99sbildn4pd in our ranking^27^. Additionally, they also observed that c22s3p and a03ws overestimates the α-helix propensity (Fig. 4).

Additionally, our results are consistent with Piana et al., who tested three and four-site water models with AMBER and CHARMM FFs^42^. Where using the TIP3P (3-site) water model, CHARMM FFs were found to be more flexible than AMBER FFs, and when using 4-site water models, AMBER FFs became a lot more flexible, except for a99sb4pew (Fig. S4).

In this study, we investigated the effectiveness of force fields developed for globular proteins and their modified versions for intrinsically disordered proteins. We found that these force fields, with their adapted water models, produce distinct conformational ensembles. Our results highlight the importance of utilizing multiple measures for a comprehensive evaluation of force fields due to the intrinsic flexibility of this system which can form β-strand fibrils and adopt random coiled conformations.

Our evaluation of thirteen force fields from CHARMM and AMBER families revealed that c36m2021s3p is the most balanced in terms of the three measures used. This force field can generate various conformations that are compatible with U/L-shaped conformations, and it showed good agreement in terms of the SSP and intra-peptide contact map. Additionally, its mTIP3P water model is computationally less expensive than those of top-ranked AMBER FFs with four-site water models.

## Methods

### Preparation of initial conformations for molecular dynamics simulations

Multiple 3D-structures of FUS-LC domain have been solved by X-ray crystallography^17,51^, electron crystallography^17,52^, cryo-Electron Microscopy (cryo-EM)^24^, and NMR^16^. The details of the structures are listed in the Table S5.

From the 20 U-shaped NMR models (PDB ID: 5W3N^16^), the region of residues 50–65 (R2-FUS-LC region, Fig. 1, red square) of the first three chains (trimer) was used. The 20 structures were clustered into six groups using the GROMACS tool *gmx cluster*^53^ with a RMSD cutoff of 1.7 Å (Cα-only). The six representative structures were used to run six independent MD simulations.

### Molecular dynamics simulations

#### Systems

All-atom MD simulations were performed using GROMACS 2020.4^54,55^. The six initial conformations of R2-FUS-LC (663 atoms) were solvated in a cubic box of 80 × 80 × 80 Å^3^. To replicate the conditions of the NMR experiment^22^, 137 mM NaCl ions were added (~ 17,000 water molecules). The final systems contain 52,000–68,000 atoms.

#### Force fields

The 13 IDP force fields (FFs) and their corresponding water models (WMs) (details in Table S1 and Sup. Info. Section Benchmark of Force Fields (FFs) and Water Models (WMs)): a03ws, a14sb3p, a19sbopc, a99disp, a99sb4pew, a99sbCufix3p, a99sbildn4pd, c22s3p, c27s3p, c36m2021s3p, c36m2021s3pm, c36m3pm and c36ms3p.

#### Molecular dynamics simulations

Non-bonded cutoff is set to 12 Å. Electrostatic interactions were treated with the smooth particle mesh Ewald method^56^ for long range interactions and Coulomb for short range interactions. For CHARMM FFs, the Lennard–Jones potential is modified by GROMAC’s force-switching function^54,55^ between 8 and 12 Å.

The length of solute and water covalent bonds involving hydrogen atoms were kept constant using the LINCS^57^ and SETTLE^58^ algorithms, respectively, allowing integration of equations of motion with a 2 fs time step.

For each system, energy minimization followed by a constant pressure and temperature (NPT) 1 ns equilibration run were performed at 1 bar and 300 K. Temperature is controlled by the v-rescale thermostat and pressure by the Berendsen barostat^59,60^ with the time coupling constants τ_T_ = 0.1 ps and τ_P_ = 0.5 ps, respectively. A second equilibration run was performed with Nose–Hoover thermostat and Parrinello–Rahman barostat^61–63^ with the time coupling constants τ_T_ = 0.5 ps and τ_P_ = 2.5 ps for 1 ns.

For the production run, each of the six systems were simulated for 500 ns under the same conditions as in the second equilibration run but with a velocity random seed. Snapshots were saved every 20 ps. The first 100 ns of the production run were discarded, and the remaining 400 ns was used for further analysis.

The above protocol was applied to each of the 13 force fields, giving an accumulated trajectory of 3 μs.

### Data analysis

Analysis of snapshots were performed with MDAnalysis^64,65^, MDtraj^66^, DSSP^46,47^ and our own scripts. All figures were plotted with Matplotlib^44^ (Python module).

#### Normalization of raw scores

All the raw scores were linearly rescaled such that they are between 0.0001 and 1 by:

1 $${S}_{norm,\mathrm{FF}}=\frac{{S}_{raw,\mathrm{FF}}}{\underset{\mathit{FF}}{\mathrm{max}}{S}_{raw,\mathrm{FF}}-\underset{\mathit{FF}}{\mathrm{min}}{S}_{raw,\mathrm{FF}}}$$

where ${S}_{norm,\mathrm{FF}}$ and ${S}_{raw,\mathrm{FF}}$ are the normalized and raw scores for a specific force field (FF), respectively.

#### Radius of gyration

For each snapshot, the radius of gyration (Rg) of the heavy-atoms of the 16-residue trimer and the individual peptides were computed with MDAnalysis.

The Rg distribution of the trimer (Fig. 2 and Fig. S4) was fitted to two Gaussians using the gaussian mixture model.

To evaluate the FFs, the ${\text{Rg-Score}}_{FF,k}$ (raw score) compares the simulation results to the experimental ${Rg}_{exp,k}$ of *k* (U-shaped [averaged over 20 models]: 10 Å or L-shaped: 14.4 Å). It is computed as:

$${\text{Z-score}}_{FF,k,i}=\frac{{Rg}_{exp,k}-\langle {Rg}_{FF,i}\rangle }{{SD}_{FF,i}}$$

2 $${\text{Rg-Score}}_{FF,k}=\frac{1}{\underset{i}{min}\left|{\text{Z-score}}_{FF,k,i}\right|}$$

where $\langle {Rg}_{FF,i}\rangle$ and ${SD}_{FF,i}$ are the average and standard deviation, respectively, of the *i*’th Gaussian.

The Rg of the individual peptides was compared to the predicted $R{g}_{FL}$ (10.8 Å) from Flory’s polymer theory with parameters optimized for IDPs by Bernado and Blackledge^43^:

$$R{g}_{FL}={R}_{0}\times {N}^{v}$$

3 $${\text{Unfolded Rg-Score}}_{\mathrm{FF}}= \frac{q3-q1}{\left|R{g}_{FL}- R{g}_{FF,med}\right|}$$

where ${R}_{0}=$ 2.54 Å is the persistence length, ν = 0.522 is the exponential scaling factor, N is the number of residues, $R{g}_{FF,med}$ is the median Rg of the FF and q3 and q1 are the 75th and 25th percentile, respectively.

The raw scores from (2) and (3) were normalized. The final Rg score is the normalized product of the normalized U-shaped, L-shaped, and Unfolded Rg (Table 2).

#### Intra-peptide contact map analysis

To analyze the heavy atom contacts in each peptide, we developed custom code to generate contact maps. A contact was defined as two atoms being within a 5 Å distance, except for neighboring residues along the protein sequence. We counted intra-peptide contacts across all snapshots and filtered out contact frequencies < 1%. Average contact frequencies were then calculated across all three peptides.

Comparing the average contact map from each snapshot to the representative U-shaped conformation (Fig. 3), contacts were classified into one of four groups [Table 6; True(T)/False(F) Positive(P)/Negative(N)]. A contact (no contact) is considered positive (negative). The contacts from all snapshots of the six replicas were accumulated in the confusion matrix (Table S2).

**Table 6:** General confusion matrix.

Total population		Contacts in the U-shaped reference structure	
		Positive (P)	Negative (N)
Contacts in the trajectory	Positive (PP)	True positive (TP)	False positive (FP)
	Negative (PN)	False negative (FN)	True negative (TN)

The Matthews correlation coefficient (MCC)^45^ was used to measure the agreement of the contact maps:

4 $$\mathrm{MCC}=\frac{\left(TP*TN\right)- \left(FP*FN\right)}{\sqrt{\left(\mathrm{TP}+\mathrm{FP}\right)\left(TP+FN\right)\left(TN+FP\right)\left(\mathrm{TN}+\mathrm{FN}\right)}}$$

Note that the MCC score is between − 1 and 1. An MCC score of 1 shows perfect correlation with the reference, 0 with no correlation and − 1 with perfectly negative correlation. The raw MCC score is then normalized to obtain the normalized intra-peptide Contact Map score (Table 3).

#### Inter-peptide contact map analysis

We classified the peptide's conformation in each snapshot as monomer, dimer, or trimer based on their contacts with other peptides (Table S4). The monomer was defined as having no contact with other peptides, while the dimer had exactly two peptides in contact. The trimer was characterized by each peptide being in contact with another peptide. Two peptides are considered in contact if they have at least one contact. We use the same contact definition as in the intra-peptide analysis.

To identify the middle peptide in trimers, we determined the peptide with the highest inter-peptide contacts in the fibril. We then compared the computed contact map with the experimental contact map of the middle peptide with one of the other two peptides.

For experimental structures, the contact maps of the middle peptide with either of the edge peptides were similar so we chose chain A-B for comparison with the computed contact maps (see Fig. S7). The same contact map was used for dimers.

All contacts between two edge peptides in the ensembles were considered false positives (FPs). We employed the same method as the previous section to classify contacts and compute Matthews Correlation Coefficient (MCC) scores (Table S3).

#### Secondary structure propensity

To assign secondary structures, we utilized the Dictionary of Protein Secondary Structure (DSSP)^46,47^. Each residue was classified as either α-helix (H), β-strand (E), or coil (C).

Each residue’s secondary structure propensity/probability (${p}_{i,ss}$, SSP) is computed from the snapshots, combining the data from all three peptides from the same FF:

5 $${p}_{i,ss}=\frac{{\sum }_{j,t}s{s}_{i,j,t}}{{\sum }_{ss=H,E,C}{\sum }_{i,j,t}s{s}_{i,j,t}}$$

where: *i* is the residue position in peptide *j* at snapshot *t* from the simulation performed with the forcefield. *ss*_*i,j,t*_ is 1 when assigned the respective secondary structure type *ss* by DSSP or 0 otherwise. *ss* represents the secondary structure type: α-helix (H), β-strand (E), or coil (C).

The reference secondary structures were obtained from the U-shaped (20 models) and L-shaped (single structure) structures, as shown in Table S6. The FF’s secondary structure score with respect to the experimental structure *k* (U/L-shaped) is:

6 $${\text{SSP-Score}}_{k}={\sum }_{i=1}^{{N}_{res}}\mathrm{log}{p}_{i,ss={exptl}_{k,i}}$$

where, *i* represents the residue position, and ${exptl}_{k,i}$ is the secondary structure of residue *i* in the experimental structure *k*.

For each FF, the SSP-Score is the log likelihood of observing the experimental secondary structures. We derived the probabilities from the observed propensities from the simulations, assuming that the positions are independent of one another.

The raw FF’s SSP-Scores for the U-shaped and L-shaped conformations were independently renormalized. We took the product of these two normalized SSP-scores and re-normalized them to get the final SSP score (Table 5).

### Final scoring

Using the three normalized Rg score, SSP score and intra-peptide MCC score, we calculated the final score for each FF by multiplying the scores and re-normalizing the product (Table 1).

### Supplementary Information

Supplementary Information.

## Data Availability

Data available on reasonable request to HK.
