# Supplementary material for: Benchmarking of force fields to characterize the intrinsically disordered R2-FUS-LC region
Source: Sci Rep. 2023 Aug 30;13:14226. doi: 10.1038/s41598-023-40801-6 (PMC10468508; doi:10.1038/s41598-023-40801-6)
Supplement: Supplementary file 1 — Supplementary Information. [file 41598_2023_40801_MOESM1_ESM.docx]

**SUPPORTING INFORMATION**

**Benchmarking of Force Fields to Characterize the Intrinsically Disordered R2-FUS-LC Region**

**Maud Chan-Yao-Chong^1,2^, Justin Chan Wai Soon^1^, Hidetoshi Kono*^1^**

1. Molecular Modeling and Simulation (MMS) Team, Institute for Quantum Life Science, National Institutes for Quantum Science and Technology (QST) 4-9-1, Anagawa, Inage ward, Chiba city, Chiba, 263-8555 Japan
2. Toulouse Biotechnology Institute, TBI, Université de Toulouse, CNRS, INRAE, INSA, Toulouse, France. 135, avenue de Rangueil, F-31077 Toulouse Cedex 04, France.

E-mail: kono.hidetoshi@qst.go.jp

tel:+81-43-382-4295

**Benchmark of Force Fields (FFs) and Water Models (WMs)**

In the late 20th century, all-atom protein FFs were developed to simulate the conformational dynamics of folded proteins (AMBER99^1^, AMBER14^2^, CHARMM22^3^ with the three-site water model, SPC/E^4^ or sTIP3P^5^), which generally have their nonpolar residues buried in their core and protected from solvent. This characteristic is observed only for globular and folded protein but not for IDPs / IDRs.

That is why, recently, several FF / WM were improved to properly generate conformational ensembles of IDPs / IDRs, in which nonpolar residues are frequently exposed to solvent while being suitable for globular proteins.

One approach is to modify the protein-water interaction at level of water model:

This is generally achieved by using a four-site water model, such as TIP4P^5^ or OPC water model^6^, which better accounts for the electric properties of water molecules, and by accentuating the depth of the solute-solvent Lennard-Jones (LJ) potentials to better solvate nonpolar residues.

TIP4P water model reparametrized with Ewald techniques provide the new water model TIP4P-Ew in 2004^7^.

The second improvement was reported by Best et al., who proposed to rescale by a factor γ = 1.1 the LJ parameters εOi between the water oxygen of model TIP4P/2005 and the protein atoms of AMBER-03w FF^8^. This modified water-solute interactions was denoted in the literature as TIP4P/2005s. The combination of AMBER-03w with TIP4P/2005s is named A03ws.

Another improvement, to counter the underestimation of water dispersion interaction with some polar amino acid, is to increase the Lennard-Jones dispersion coefficient C_6_ = 900 kcal mol^-1^ Å^6^, for TIP4P/2005 and called the new water model TIP4P-D^9^.

The first improvement from standard TIP3P (sTIP3P)^5^ to CHARMM-TIP3P (mTIP3P)^3^ was to add the ε_H_ = -0.046 kcal mol^-1^ and the σ_H_ = 0.040 nm of the water hydrogen and the non-bonded interaction, and the ε_OH_ = -0.0836 kcal mol^-1^ and the σ_OH_ = 0.177 nm between the water hydrogen and the water oxygen.

One improvement concerns the CHARMM FF by increase the Lennard-Jones well depth parameter ε_H_ = -0.10 kcal mol^-1^ of the water hydrogen of the specific mTIP3P water model, this water model named CHARMM-TIP3Pm (mTIP3Pm)^10^.

The second approach is to modify this protein-water interaction at level of FF:

Another improvement concerns the AMBER FF. Yoo and Aksimentiev optimized the AMBER99SB-ILDN-Phi FF with their pair-specific Lennard-Jones parameters describing amine nitrogen-carboxylate oxygen interactions and also aliphatic carbon-carbon pairs thanks to experimental data of osmotic pressure from amino acid in solution, named this new FF AMBER99SB-ILDN-phi-CUFIX (A99Cufix3p)^11^.

http://bionano.physics.illinois.edu/CUFIX

The third approach is to modify the dihedral parameters:

This parameter modifies the energy of the system as a function of rotation around bonds. Since the flexibility of the biomolecule is correlated to the bond rotations, these corrections can affect the sampling of the conformational ensemble of the studied biomolecule.

The improvement for the FF was first focused on the backbone φ and ψ dihedral potentials to better reproduce the two-dimensional Ramachandran probability distributions like for AMBER03w^12^, CHARMM22*^13^, AMBER14SB^2^, AMBER19SB^14^ FFs. AMBER19SB is the updated FF of AMBER99SB and AMBER14SB.

Another optimization improved sides chain torsion potentials to refine side-chain rotamers to improve the helix propensities, calling ‘-ILDN’ extension of some FFs, like AMBER99SB-ILDN^15,16^.

From this idea, a fourth approach is to optimize grid-based energy corrections maps (CMAP potential):

MacKerell et al. optimized CHARMM22 with correction maps based on cross-term energy functions of φ and ψ angles to better account for their correlations (CMAP) based on experimental NMR observables for backbone. This new FF is called CHARMM27^3,17^.

MacKerell et al. goes even further by updating this CMAP potentials to correct the φ and ψ probability distributions for intrinsically disordered proteins, optimizing the side-chain dihedral potentials from IDP’s NMR data^18^, and updating from CAMP strategy and divided Cα into 3 different groups in function of the residue type. These modifications conduct to update CHARMM27 to CHARMM36 and to get CHARMM36m^10^.

Every year, some small updates for CHARMM36m for proteins-ions-lipids were adjusted. The last update used was in February 2021 where the NBFIX terms have been changed between chloride and sodium ions interaction.


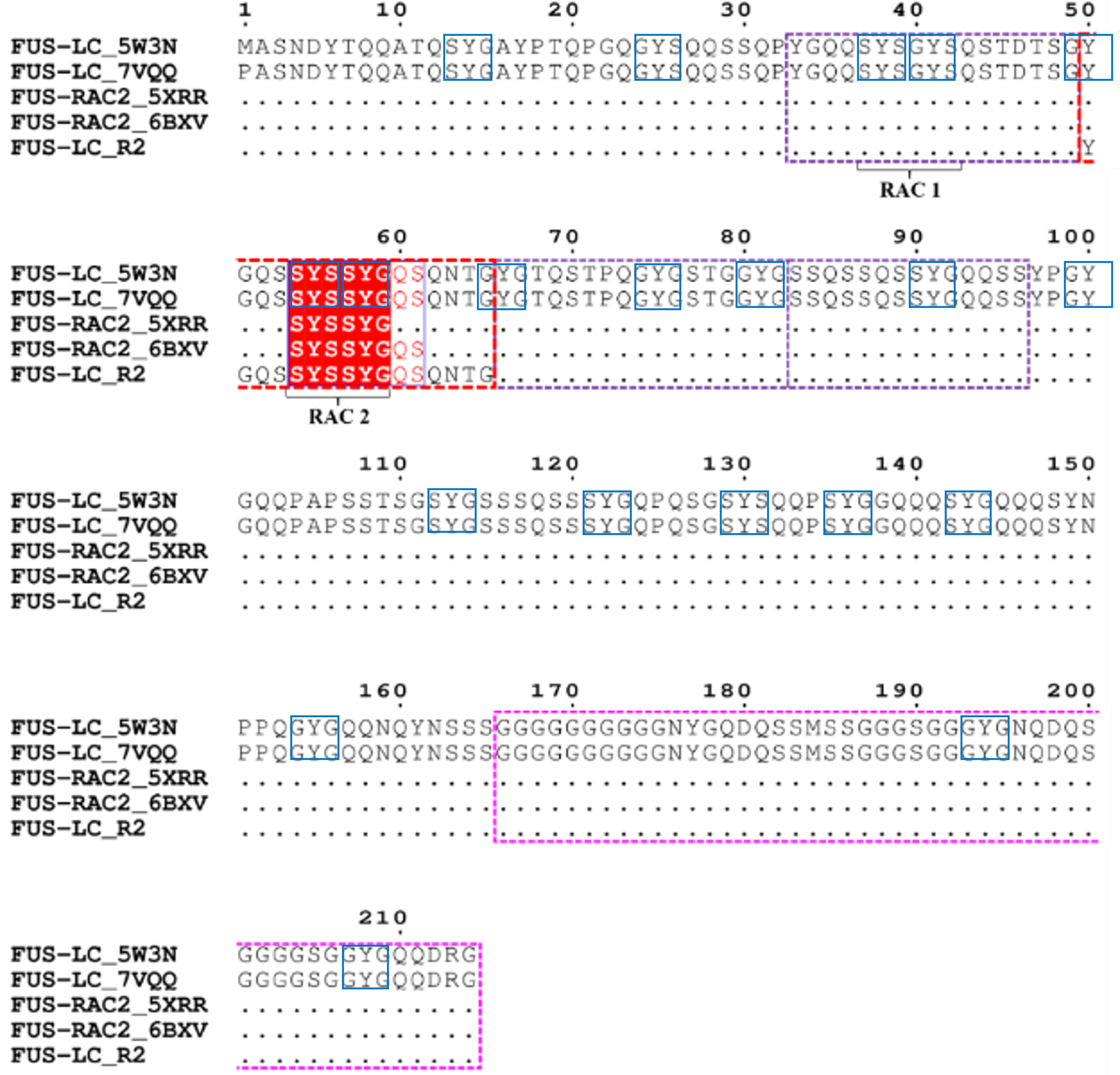


**Figure S1.** **Sequence of the full-length FUS-LC_1-214_ domain.** Multiple sequence alignment of the human full-length Low-Complexity (LC) domain of FUS protein used by Murray *et al.* (FUS_5W3N, PDB ID: 5W3N). The amino acid numbering is based on the human protein sequence (UNIPROT ID: P35637). The 20 blue boxes correspond to the motif of three residues [S/G]Y[S/G]. The red box represents the R2 region of the FUS-LC domain (R2-FUS-LC region) containing the Reversible Amyloid Cores (RAC2) residues highlighted in red. The three violet boxes indicate the repeat region R1 (containing RAC1), R3 and R4, respectively. The pink box indicates the Gly-rich domain. Figure was prepared with Microsoft PowerPoint and UCSF Chimera v1.11.2^19,20^ (<https://www.cgl.ucsf.edu/chimera/>).

**
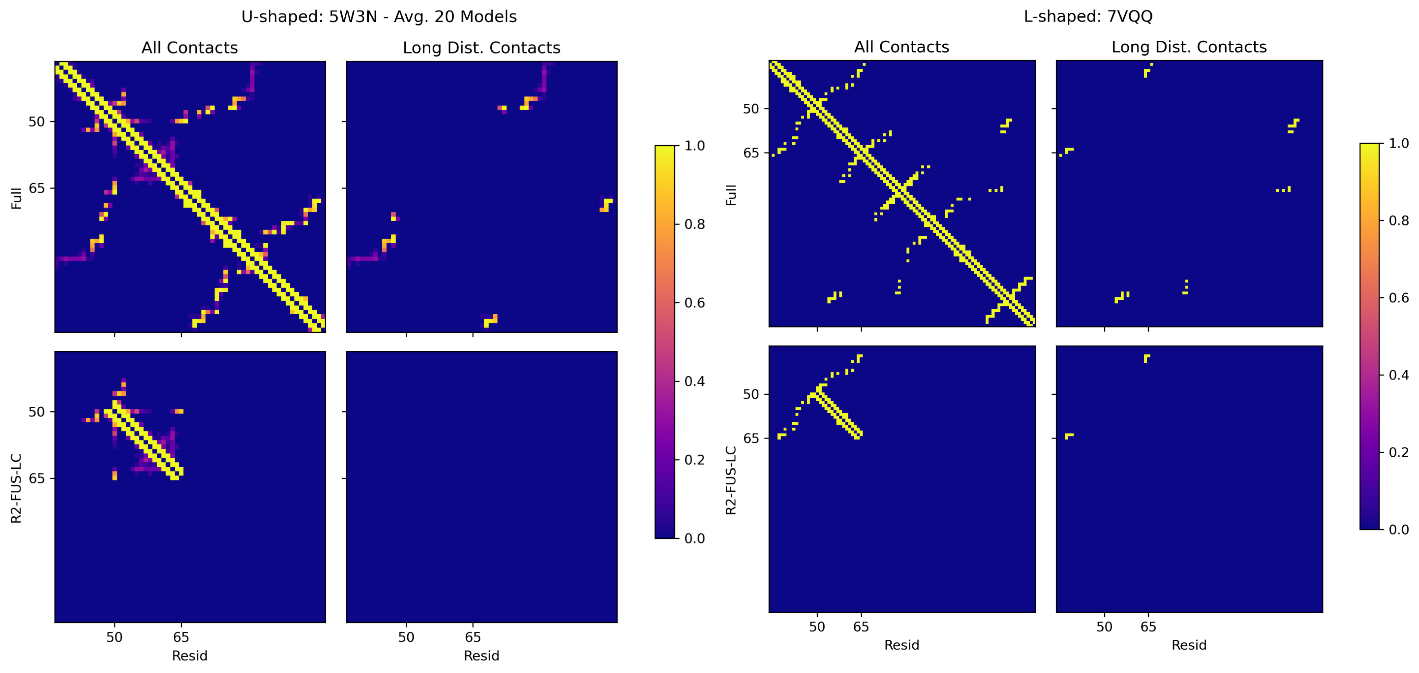
**

**Figure S2: Full-length FUS-LC_1-214_ domain intra-contact analysis*.*** Contact maps of the intra-protein interaction for the full-length U-shaped (PDB ID: 5W3N) and L-shaped (PDB ID: 7VQQ) conformations (top left panels). The number of long-distance contacts (top right panels, contacts that are ≥15 residues away) are few. The long-distance contacts between the R2-FUS-LC region (residues 50-65) with the rest of the protein is shown in the bottom right panels. In the U-shaped conformation, there are no long-distance contacts while there are only a few in the L-shaped conformation. Figure was prepared with Matplotlib v3.5^21^ (https://matplotlib.org/).

**
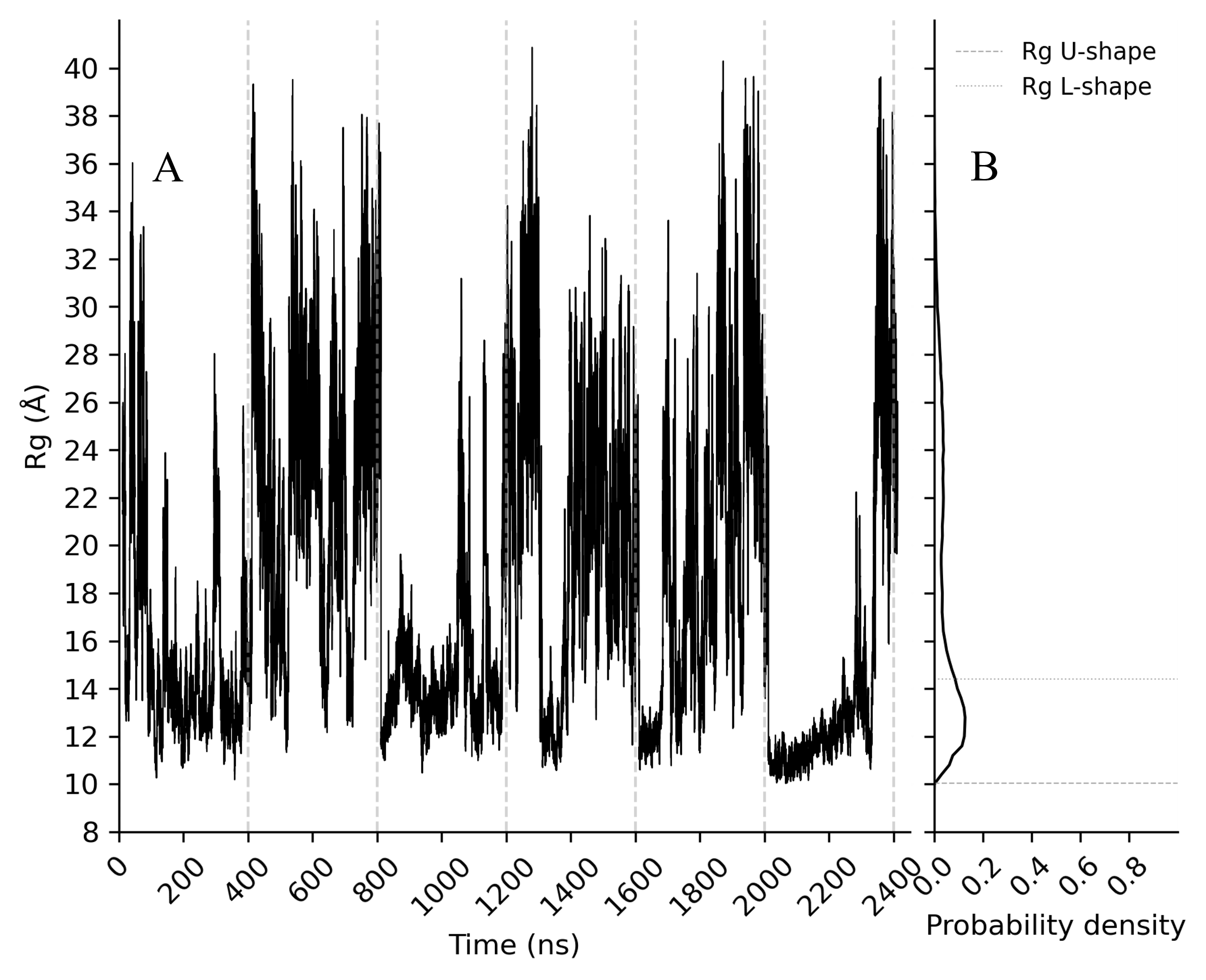
**

**Figure S3. Radius of gyration (Rg) in function of trajectory times (A) and distribution of the Rg (B) for c36m2021s3p FF.** Figure was prepared with Matplotlib v3.5^21^ (https://matplotlib.org/).


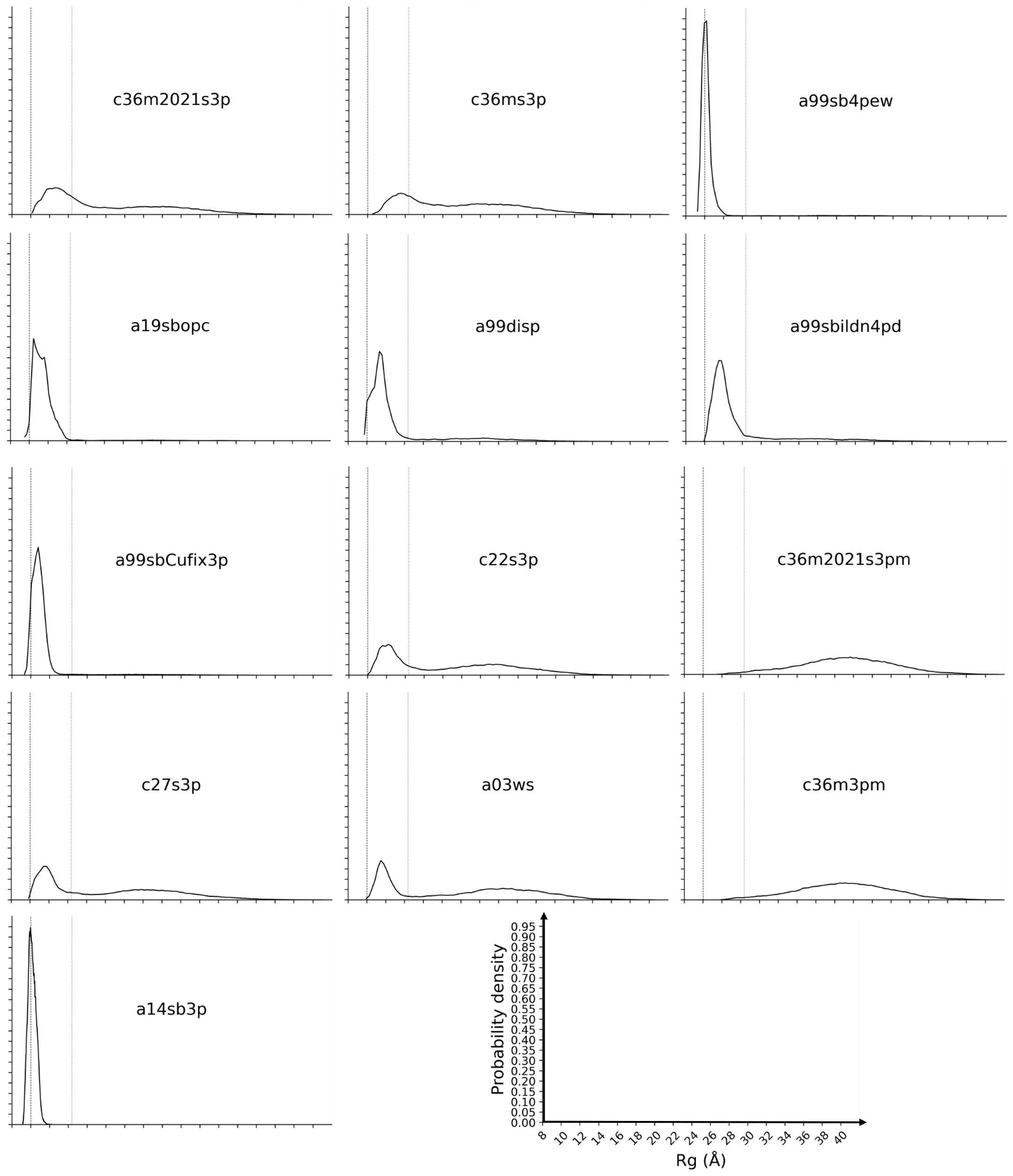


**Figure S4. Distribution of the Radius of gyration (Rg) for the AMBER and the CHARMM FFs.** Vertical dash and dot lines correspond to the average of the Rg for U-shape (10.0 Å) and L-shape (14.4 Å), respectively. Figure was prepared with Matplotlib v3.5^21^ (https://matplotlib.org/).


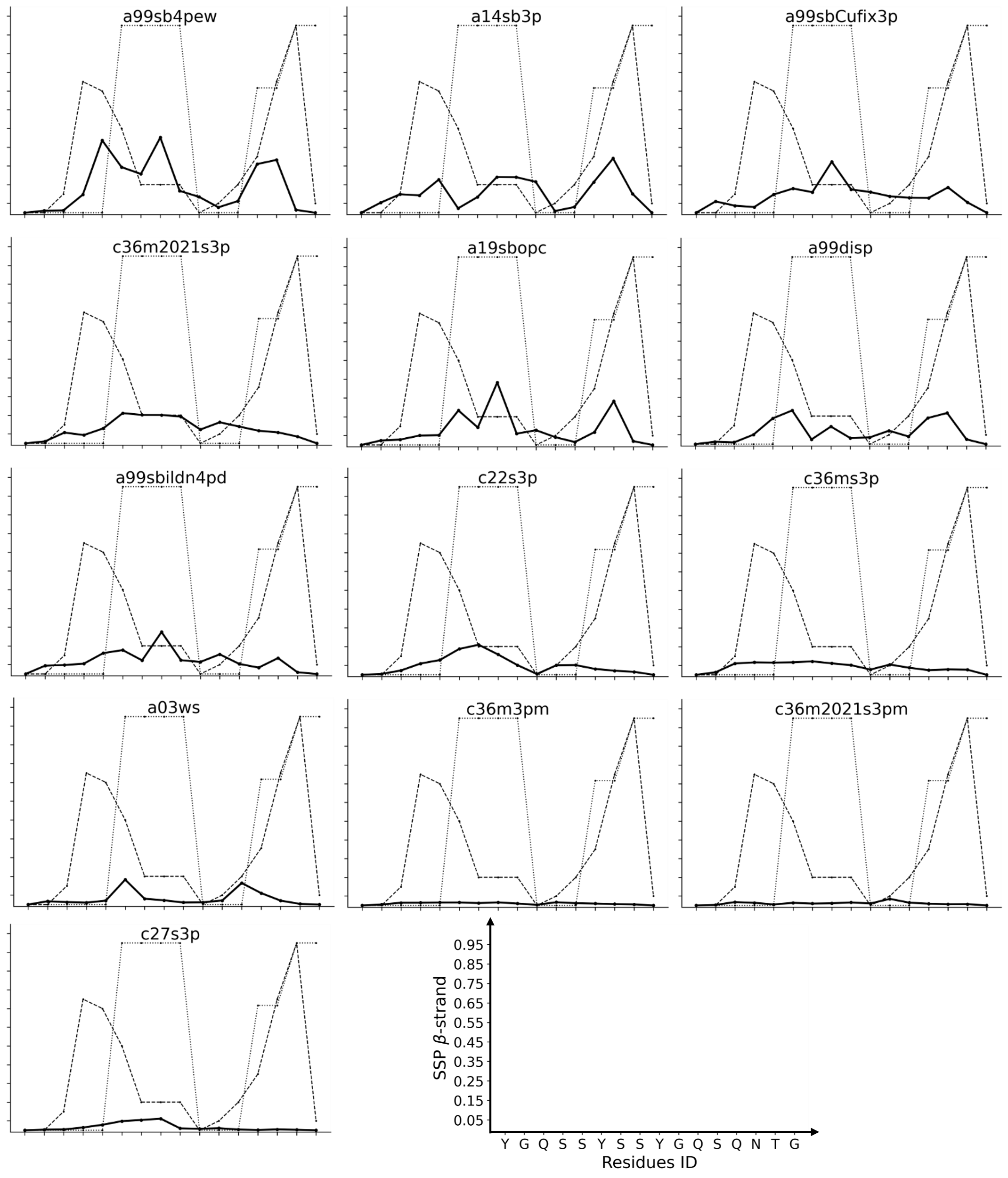


**Figure S5. β-strand Secondary Structure Propensity (SSP)** for thirteen FFs (solid line), dash line and dot line correspond to the β-strand SSP of U-shape and L-shape, respectively. Figure was prepared with Matplotlib v3.5^21^ (https://matplotlib.org/).


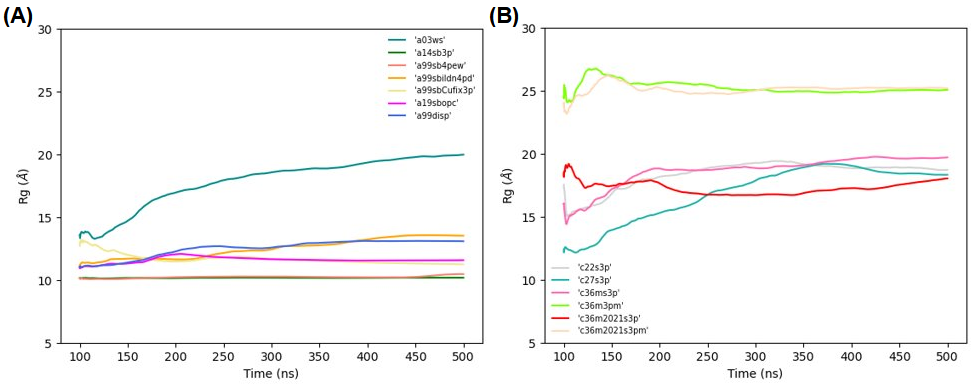


**Figure S6.** **Convergence of Rg - Average** **Rg vs Time**. Accumulated average Rg from 100 ns to time t for **(A)** AMBER FFs **(B)** CHARMM FFs. Except for a03ws and c27s3p, all FFs nearly converge within 300 ns. Figure was prepared with Matplotlib v3.5^21^ (https://matplotlib.org/).


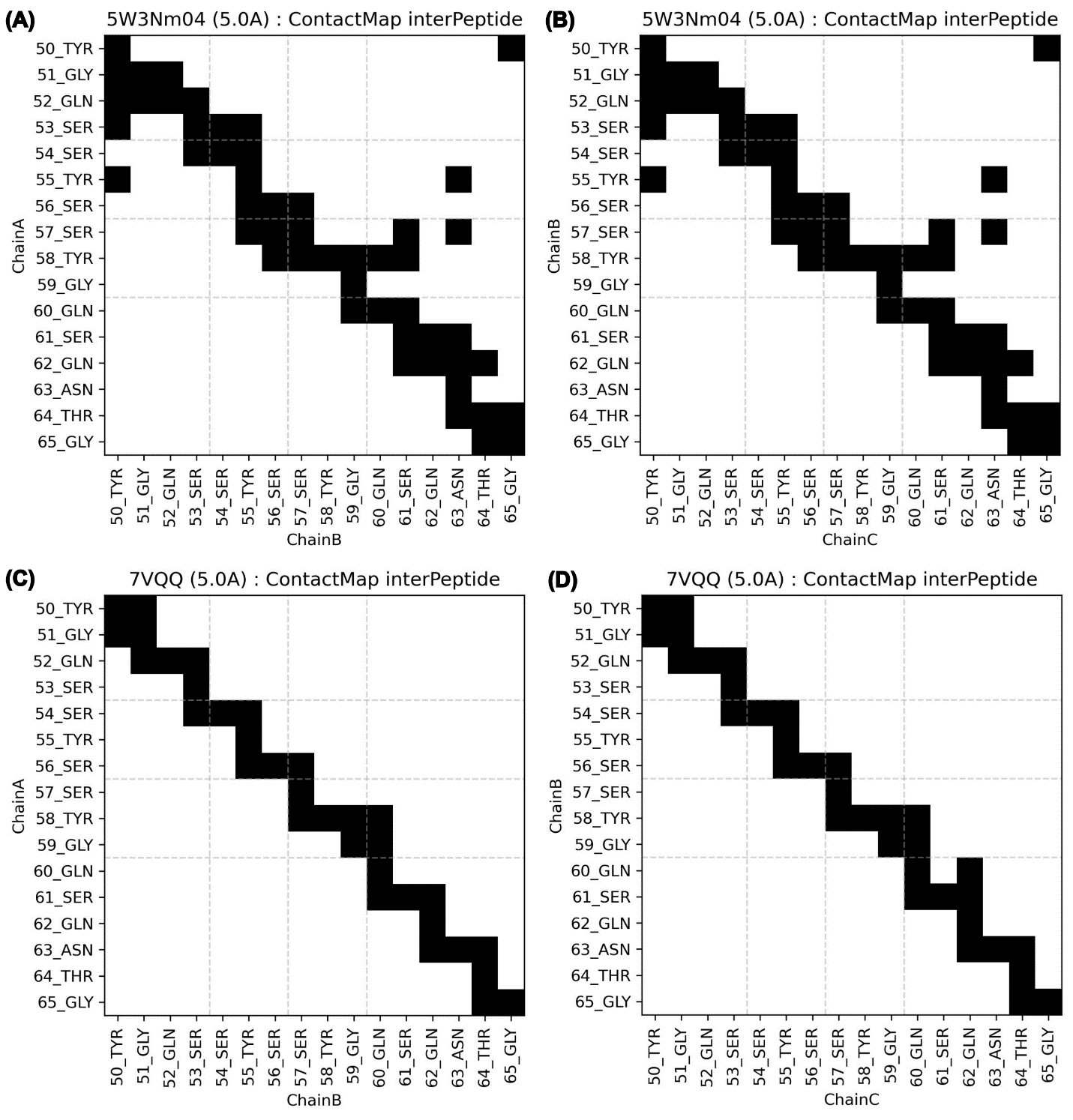


**Figure S7.** **Experimental Inter-chain** **Contact Maps Between Peptide Chains.** Chain B is sandwiched between Chain A and C. Chains A and C have no contacts with one another. **(A-B)** From the U-shaped conformation. **(C-D)** From the L-shaped conformation. Figure was prepared with Matplotlib v3.5^21^ (https://matplotlib.org/).

**Table S1. Description of Force Fields (FFs) and Water Models (WMs).**

| **FF (Year)** | **Modifications** | **WM (Year)** | **Modifications** | **Abbreviation** |
| --- | --- | --- | --- | --- |
| AMBER99SB (2006)^22^ |  | TIP4P-Ew (2004)^7^ | Protein-water interaction | A99sb4pew |
| AMBER03w (2010)^12^ | Dihedral parameters (backbone) | TIP4P/2005s (2014)^8^ | Protein-water interaction | A03ws |
| AMBER99SB-ILDN (2010)^15^ | Protein-water interaction | TIP4P-D (2015)^9^ | Protein-water interaction | A99sbildn4pd |
| AMBER14SB (2015)^2^ | Dihedral parameters (Backbone) | TIP3P (1983)^5^ |  | A14sb3p |
| AMBER99SB-ILDN-phi-Cufix (2016)^11^ | Protein-water interaction (Nonbonded fix strategy NBFIX) + Dihedral parameters (sidechain) | TIP3P (1983)^5^ |  | A99Cufix3p |
| AMBER19SB (2020)^14^ | Dihedral parameters to improve helical propensities of A14SB (Backbone profiles for all 20 amino acids) | OPC (2014)^6^ | Not parameterized against a specific atomistic WM for IDP | A19sbopc |
| AMBER99SB-Disp (2018)^23^ | Optimized a99SB-ILDN by changing the protein backbone hydrogen bonds interaction | A99SB-disp water^23^ | Optimized TIP4P-D by increasing the C6 dispersion term to avoid the over collapse the helical propensity | A99disp |
| CHARMM27 (2004)^3,17^ | CMAP potential | mTIP3P (1998)^3,5^ | Protein-water interaction (ε/σ_H_ and ε/σ_OH_) | C27s3p |
| CHARMM22* (2011)^3,13^ | Dihedral parameters (Backbone and sidechain) | mTIP3P (1998)^3,5^ | Protein-water interaction (ε/σ_H_ and ε/σ_OH_) | C22s3p |
| CHARMM36m (2017)^10^ | CMAP potential (based on NMR data from IDPs) | mTIP3P (1998)^3,5^ | Protein-water interaction (ε/σ_H_ and ε/σ_OH_) | C36ms3p |
| CHARMM36m (2017)^10^ | CMAP potential (based on NMR data from IDPs) | mTIP3Pm (2016)^10^ | Protein-water interaction (ε_H_) for IDP | C36m3pm |
| CHARMM36m2021 (2021)^10^ | CMAP potential (based on NMR data from IDPs) | mTIP3P (1998)^3,5^ | Protein-water interaction (ε/σ_H_ and ε/σ_OH_) | C36m2021s3p |
| CHARMM36m2021 (2021)^10^ | CMAP potential (based on NMR data from IDPs) | mTIP3Pm (2016)^10^ | Protein-water interaction (ε_H_) for IDP | C36m2021s3pm |

**Table S2. Confusion matrix of the 13 FFs.**

| **c36m2021s3pm^*^** | **Ref_T** | **Ref_F** |  | **a99disp^*^** | **Ref_T** | **Ref_F** |
| --- | --- | --- | --- | --- | --- | --- |
| **Pred_T** | 13.75 | 3.52 |  | **Pred_T** | 14.07 | 7.18 |
| **Pred_F** | 5.30 | 77.43 |  | **Pred_F** | 4.98 | 73.77 |
|  |  |  |  |  |  |  |
| **c36m3pm^#^** | **Ref_T** | **Ref_F** |  | **c22s3p^*^** | **Ref_T** | **Ref_F** |
| **Pred_T** | 13.75 | 3.57 |  | **Pred_T** | 14.02 | 7.16 |
| **Pred_F** | 5.29 | 77.38 |  | **Pred_F** | 5.03 | 73.80 |
|  |  |  |  |  |  |  |
| **c36ms3p^*^** | **Ref_T** | **Ref_F** |  | **a99sbildn4pd^*^** | **Ref_T** | **Ref_F** |
| **Pred_T** | 13.86 | 4.61 |  | **Pred_T** | 14.16 | 7.88 |
| **Pred_F** | 5.19 | 76.34 |  | **Pred_F** | 4.88 | 73.07 |
|  |  |  |  |  |  |  |
| **c36m2021s3p^*^** | **Ref_T** | **Ref_F** |  | **a14sb3p^#^** | **Ref_T** | **Ref_F** |
| **Pred_T** | 13.86 | 5.13 |  | **Pred_T** | 14.30 | 9.18 |
| **Pred_F** | 5.18 | 75.82 |  | **Pred_F** | 4.43 | 72.09 |
|  |  |  |  |  |  |  |
| **a19sbopc^*^** | **Ref_T** | **Ref_F** |  | **a99sbCufix3p^•^** | **Ref_T** | **Ref_F** |
| **Pred_T** | 14.02 | 6.16 |  | **Pred_T** | 14.17 | 8.99 |
| **Pred_F** | 4.71 | 75.11 |  | **Pred_F** | 4.88 | 71.96 |
|  |  |  |  |  |  |  |
| **a99sb4pew^*^** | **Ref_T** | **Ref_F** |  | **a03ws^#^** | **Ref_T** | **Ref_F** |
| **Pred_T** | 14.84 | 7.71 |  | **Pred_T** | 13.98 | 10.82 |
| **Pred_F** | 4.21 | 73.25 |  | **Pred_F** | 5.06 | 70.13 |
|  |  |  |  |  |  |  |
| **Confusion Matrix** | **Ref_T** | **Ref_F** |  | **c27s3p^#^** | **Ref_T** | **Ref_F** |
| **Pred_T** | TP | FP |  | **Pred_T** | 14.50 | 12.98 |
| **Pred_F** | FN | TN |  | **Pred_F** | 4.55 | 67.97 |

**Table S3: Matthews Correlation Coefficient Score (MCC Score)** **for the inter-chain interaction**.

| FFs | MCC Score  U-shaped | MCC Score  L-shaped |
| --- | --- | --- |
| a14sb3p | 0.02136 | 0.01982 |
| a19sbopc | 0.02131 | 0.01982 |
| a99sbCufix3p | 0.02020 | 0.01913 |
| a99sbildn4pd | 0.01895 | 0.01797 |
| a99sb4pew | 0.01860 | 0.01728 |
| a03ws | 0.01818 | 0.01661 |
| c27s3p | 0.01555 | 0.01363 |
| a99disp | 0.01537 | 0.01442 |
| c22s3p | 0.01268 | 0.01057 |
| c36m2021s3pm | 0.01137 | 0.01340 |
| c36m2021s3p | 0.00823 | 0.00726 |
| c36m3pm | 0.00629 | 0.01006 |
| c36ms3p | 0.00600 | 0.00643 |

**Table S4: Monomer, Dimer and Trimer ratio of ensembles obtained with the 13 FFs.**

| FFs | Monomer Ratio (%) | Dimer Ratio (%) | Trimer Ratio (%) |
| --- | --- | --- | --- |
| a03ws | 31.76 | 28.80 | 39.44 |
| a14sb3p | 0.00 | 0.00 | 100.00 |
| a19sbopc | 0.00 | 1.97 | 98.03 |
| a99disp | 2.01 | 12.22 | 85.78 |
| a99sb4pew | 0.00 | 2.20 | 97.80 |
| a99sbCufix3p | 0.00* | 3.53 | 96.47 |
| a99sbildn4pd | 0.97 | 13.66 | 85.38 |
| c22s3p | 13.76 | 40.12 | 46.13 |
| c27s3p | 16.14 | 38.55 | 45.31 |
| c36ms3p | 12.84 | 42.41 | 44.75 |
| c36m3pm | 44.40 | 47.07 | 8.54 |
| c36m2021s3p | 12.25 | 30.76 | 56.99 |
| c36m2021s3pm | 48.70 | 41.03 | 10.27 |

* For a99sbCufix3p, the ratio for the monomer is 0.0025

**Table S5.** **List of PDB structures from FUS-LC domain or short region of this domain.** *: PDB ID with R2-FUS-LC region solved or partially solved.

| **PDB ID** | **Year** | **Method** | **Purified Protein residues** | **Visible residues** | **Publication** |
| --- | --- | --- | --- | --- | --- |
| **5W3N*** | **2017** | **NMR** | **2 - 214** | **37 – 97**  **(20 models)** | ^24^ |
| 5XSG | 2018 | Electron Crystallography (0.73 Å) | 37 – 42 | 37-42 | ^25^ |
| **5XRR*** | 2018 | X-ray (1.5 Å) | 54 - 59 | **54 - 59** | ^25^ |
| 6BWZ | 2018 | X-ray (1.1 Å) | 37-42 | 37-42 | ^26^ |
| **6BXV*** | 2018 | X-ray (1.1 Å) | 54 - 61 | **54 - 61** | ^26^ |
| 6KJ1 | 2019 | Electron Crystallography (0.65 Å) | 37-42 | 37-42 | ^27^ |
| 6KJ2 | 2019 | Electron Crystallography (0.67 Å) | 37-42 | 37-42 | ^27^ |
| 6KJ3 | 2019 | Electron Crystallography (0.60 Å) | 37-42 | 37-42 | ^27^ |
| 6KJ4 | 2019 | Electron Crystallography (0.65 Å) | 37-42 | 37-42 | ^27^ |
| **7VQQ*** | **2022** | **Cryo-EM (2.90 Å)** | **2-214** | **34-124** | ^28^ |

**Table S6.** **Dictionary of Secondary Structure Propensity** (**DSSP) by residues for references L-shape and U-shape PDBs.** C, H, E correspond to Coil, Helix or strand secondary structure, respectively. The RAC2 is showing in the black square.

| **Res ID** | **Residue** | **DSSP U-shape** | **DSSP L-shape** |
| --- | --- | --- | --- |
| **50** | **TYR** | **C** | **C** |
| **51** | **GLY** | **C** | **C** |
| **52** | **GLN** | **C** | **C** |
| **53** | **SER** | **E** | **C** |
| **54** | **SER** | **E** | **C** |
| **55** | **TYR** | **E** | **E** |
| **56** | **SER** | **E** | **E** |
| **57** | **SER** | **E** | **E** |
| **58** | **TYR** | **E** | **E** |
| **59** | **GLY** | **C** | **C** |
| **60** | **GLN** | **C** | **C** |
| **61** | **SER** | **E** | **C** |
| **62** | **GLN** | **E** | **E** |
| **63** | **ASN** | **E** | **E** |
| **64** | **THR** | **E** | **E** |
| **65** | **GLY** | **C** | **E** |

(1) Wang, J.; Cieplak, P.; Kollman, P. A. How Well Does a Restrained Electrostatic Potential (RESP) Model Perform in Calculating Conformational Energies of Organic and Biological Molecules? *JOURNAL OF COMPUTATIONAL CHEMISTRY* **2000**, *21* (12), 26.

(2) Maier, J. A.; Martinez, C.; Kasavajhala, K.; Wickstrom, L.; Hauser, K. E.; Simmerling, C. Ff14SB: Improving the Accuracy of Protein Side Chain and Backbone Parameters from Ff99SB. *J. Chem. Theory Comput.* **2015**, *11* (8), 3696–3713. https://doi.org/10.1021/acs.jctc.5b00255.

(3) MacKerell, A. D.; Bashford, D.; Bellott, M.; Dunbrack, R. L.; Evanseck, J. D.; Field, M. J.; Fischer, S.; Gao, J.; Guo, H.; Ha, S.; Joseph-McCarthy, D.; Kuchnir, L.; Kuczera, K.; Lau, F. T. K.; Mattos, C.; Michnick, S.; Ngo, T.; Nguyen, D. T.; Prodhom, B.; Reiher, W. E.; Roux, B.; Schlenkrich, M.; Smith, J. C.; Stote, R.; Straub, J.; Watanabe, M.; Wiórkiewicz-Kuczera, J.; Yin, D.; Karplus, M. All-Atom Empirical Potential for Molecular Modeling and Dynamics Studies of Proteins. *J. Phys. Chem. B* **1998**, *102* (18), 3586–3616. https://doi.org/10.1021/jp973084f.

(4) Berendsen, H. J. C.; Grigera, J. R.; Straatsma, T. P. The Missing Term in Effective Pair Potentials. *The Journal of Physical Chemistry* **1987**, *91* (24), 6269–6271. https://doi.org/10.1021/j100308a038.

(5) Jorgensen, W. L.; Chandrasekhar, J.; Madura, J. D.; Impey, R. W.; Klein, M. L. Comparison of Simple Potential Functions for Simulating Liquid Water. *The Journal of Chemical Physics* **1983**, *79* (2), 926–935. https://doi.org/10.1063/1.445869.

(6) Izadi, S.; Anandakrishnan, R.; Onufriev, A. V. Building Water Models: A Different Approach. *J. Phys. Chem. Lett.* **2014**, *5* (21), 3863–3871. https://doi.org/10.1021/jz501780a.

(7) Horn, H. W.; Swope, W. C.; Pitera, J. W.; Madura, J. D.; Dick, T. J.; Hura, G. L.; Head-Gordon, T. Development of an Improved Four-Site Water Model for Biomolecular Simulations: TIP4P-Ew. *J. Chem. Phys.* **2004**, *120* (20), 9665–9678. https://doi.org/10.1063/1.1683075.

(8) Best, R. B.; Zheng, W.; Mittal, J. Balanced Protein–Water Interactions Improve Properties of Disordered Proteins and Non-Specific Protein Association. *J. Chem. Theory Comput.* **2014**, *10* (11), 5113–5124. https://doi.org/10.1021/ct500569b.

(9) Piana, S.; Donchev, A. G.; Robustelli, P.; Shaw, D. E. Water Dispersion Interactions Strongly Influence Simulated Structural Properties of Disordered Protein States. *J. Phys. Chem. B* **2015**, *119* (16), 5113–5123. https://doi.org/10.1021/jp508971m.

(10) Huang, J.; Rauscher, S.; Nawrocki, G.; Ran, T.; Feig, M.; de Groot, B. L.; Grubmüller, H.; MacKerell Jr, A. D. CHARMM36m: An Improved Force Field for Folded and Intrinsically Disordered Proteins. *Nat Meth* **2017**, *14* (1), 71–73. https://doi.org/10.1038/nmeth.4067.

(11) Yoo, J.; Aksimentiev, A. Refined Parameterization of Nonbonded Interactions Improves Conformational Sampling and Kinetics of Protein Folding Simulations. *J. Phys. Chem. Lett.* **2016**, *7* (19), 3812–3818. https://doi.org/10.1021/acs.jpclett.6b01747.

(12) Best, R. B.; Mittal, J. Protein Simulations with an Optimized Water Model: Cooperative Helix Formation and Temperature-Induced Unfolded State Collapse. *J. Phys. Chem. B* **2010**, *114* (46), 14916–14923. https://doi.org/10.1021/jp108618d.

(13) Piana, S.; Lindorff-Larsen, K.; Shaw, D. E. How Robust Are Protein Folding Simulations with Respect to Force Field Parameterization? *Biophysical Journal* **2011**, *100* (9), L47–L49. https://doi.org/10.1016/j.bpj.2011.03.051.

(14) Tian, C.; Kasavajhala, K.; Belfon, K. A. A.; Raguette, L.; Huang, H.; Migues, A. N.; Bickel, J.; Wang, Y.; Pincay, J.; Wu, Q.; Simmerling, C. Ff19SB: Amino-Acid-Specific Protein Backbone Parameters Trained against Quantum Mechanics Energy Surfaces in Solution. *J Chem Theory Comput* **2020**, *16* (1), 528–552. https://doi.org/10.1021/acs.jctc.9b00591.

(15) Lindorff-Larsen, K.; Piana, S.; Palmo, K.; Maragakis, P.; Klepeis, J. L.; Dror, R. O.; Shaw, D. E. Improved Side-Chain Torsion Potentials for the Amber Ff99SB Protein Force Field. *Proteins* **2010**, *78* (8), 1950–1958. https://doi.org/10.1002/prot.22711.

(16) Best, R. B.; de Sancho, D.; Mittal, J. Residue-Specific α-Helix Propensities from Molecular Simulation. *Biophysical Journal* **2012**, *102* (6), 1462–1467. https://doi.org/10.1016/j.bpj.2012.02.024.

(17) MacKerell, A. D.; Feig, M.; Brooks, C. L. Extending the Treatment of Backbone Energetics in Protein Force Fields: Limitations of Gas‐phase Quantum Mechanics in Reproducing Protein Conformational Distributions in Molecular Dynamics Simulations. *Journal of Computational Chemistry* **2004**, *25* (11), 1400–1415. https://doi.org/10.1002/jcc.20065.

(18) Best, R. B.; Zhu, X.; Shim, J.; Lopes, P. E. M.; Mittal, J.; Feig, M.; MacKerell, A. D. Optimization of the Additive CHARMM All-Atom Protein Force Field Targeting Improved Sampling of the Backbone φ, ψ and Side-Chain Χ1 and Χ2 Dihedral Angles. *J Chem Theory Comput* **2012**, *8* (9), 3257–3273. https://doi.org/10.1021/ct300400x.

(19) Pettersen, E. F.; Goddard, T. D.; Huang, C. C.; Couch, G. S.; Greenblatt, D. M.; Meng, E. C.; Ferrin, T. E. UCSF Chimera--a Visualization System for Exploratory Research and Analysis. *J Comput Chem* **2004**, *25* (13), 1605–1612. https://doi.org/10.1002/jcc.20084.

(20) Pei, J.; Grishin, N. V. AL2CO: Calculation of Positional Conservation in a Protein Sequence Alignment. *Bioinformatics* **2001**, *17* (8), 700–712. https://doi.org/10.1093/bioinformatics/17.8.700.

(21) Hunter, J. D. Matplotlib: A 2D Graphics Environment. *Comput. Sci. Eng.* **2007**, *9* (3), 90–95. https://doi.org/10.1109/MCSE.2007.55.

(22) Hornak, V.; Abel, R.; Okur, A.; Strockbine, B.; Roitberg, A.; Simmerling, C. Comparison of Multiple Amber Force Fields and Development of Improved Protein Backbone Parameters. *Proteins: Structure, Function, and Bioinformatics* **2006**, *65* (3), 712–725. https://doi.org/10.1002/prot.21123.

(23) Robustelli, P.; Piana, S.; Shaw, D. E. Developing a Molecular Dynamics Force Field for Both Folded and Disordered Protein States. *PNAS* **2018**, 201800690. https://doi.org/10.1073/pnas.1800690115.

(24) Murray, D. T.; Kato, M.; Lin, Y.; Thurber, K. R.; Hung, I.; McKnight, S. L.; Tycko, R. Structure of FUS Protein Fibrils and Its Relevance to Self-Assembly and Phase Separation of Low-Complexity Domains. *Cell* **2017**, *171* (3), 615-627.e16. https://doi.org/10.1016/j.cell.2017.08.048.

(25) Luo, F.; Gui, X.; Zhou, H.; Gu, J.; Li, Y.; Liu, X.; Zhao, M.; Li, D.; Li, X.; Liu, C. Atomic Structures of FUS LC Domain Segments Reveal Bases for Reversible Amyloid Fibril Formation. *Nature Structural & Molecular Biology* **2018**, *25* (4), 341–346. https://doi.org/10.1038/s41594-018-0050-8.

(26) Hughes, M. P.; Sawaya, M. R.; Boyer, D. R.; Goldschmidt, L.; Rodriguez, J. A.; Cascio, D.; Chong, L.; Gonen, T.; Eisenberg, D. S. Atomic Structures of Low-Complexity Protein Segments Reveal Kinked β Sheets That Assemble Networks. *Science* **2018**, *359* (6376), 698–701. https://doi.org/10.1126/science.aan6398.

(27) Zhou, H.; Luo, F.; Luo, Z.; Li, D.; Liu, C.; Li, X. Programming Conventional Electron Microscopes for Solving Ultrahigh-Resolution Structures of Small and Macro-Molecules. *Anal. Chem.* **2019**, *91* (17), 10996–11003. https://doi.org/10.1021/acs.analchem.9b01162.

(28) Sun, Y.; Zhang, S.; Hu, J.; Tao, Y.; Xia, W.; Gu, J.; Li, Y.; Cao, Q.; Li, D.; Liu, C. Molecular Structure of an Amyloid Fibril Formed by FUS Low-Complexity Domain. *iScience* **2022**, *25* (1), 103701. https://doi.org/10.1016/j.isci.2021.103701.
